# Previous Intensive Running or Swimming Negatively Affects CPR Effectiveness

**DOI:** 10.3390/ijerph18189843

**Published:** 2021-09-18

**Authors:** J. Arturo Abraldes, Ricardo J. Fernandes, Ricardo Morán-Navarro

**Affiliations:** 1 Research Group MS&SPORT, Faculty of Sports Sciences, University of Murcia, 30720 Murcia, Spain; abraldes@um.es; 2 Porto Biomechanics Laboratory, University of Porto, 4200-450 Porto, Portugal; 3 Centre of Research, Education, Innovation and Intervention in Sport, Faculty of Sport, University of Porto, 4200-450 Porto, Portugal; 4 Department of Physical Activity and Sport, Faculty of Sports Sciences, University of Murcia, 30720 Murcia, Spain; moran@um.es

**Keywords:** physiology, fatigue, effort, oxygen uptake, effectiveness, CPR

## Abstract

Survival outcomes increase significantly when cardiopulmonary resuscitation (CPR) is provided correctly, but rescuers’ fatigue can compromise its delivery. We investigated the effect of two exercise modes on CPR effectiveness and physiological outputs. After 4 min baseline conditions, 30 lifeguards randomly performed a 100 m run and a combined water rescue before 4 min CPR (using an adult manikin and a 30:2 compression–ventilation ratio). Physiological variables were continuously measured during baseline and CPR using a portable gas analyzer (K4b^2^, Cosmed, Rome, Italy) and CPR effectiveness was analyzed using two HD video cameras. Higher oxygen uptake (23.0 ± 9.9 and 20.6 ± 9.1 vs. 13.5 ± 6.2 mL·kg·min^−1^) and heart rate (137 ± 19 and 133 ± 15 vs. 114 ± 15 bpm), and lower compression efficacy (63.3 ± 29.5 and 62.2 ± 28.3 vs. 69.2 ± 28.0%), were found for CPRrun and CPRswim compared to CPRbase. In addition, ventilation efficacy was higher in the rescues preceded by intense exercise than in CPRbase (49.5 ± 42.3 and 51.9 ± 41.0 vs. 33.5 ± 38.3%), but no differences were detected between CPRrun and CPRswim. In conclusion, CPRrun and CPRswim protocols induced a relevant physiological stress over each min and in the overall CPR compared with CPRbase. The CPRun protocol reduces the compression rate but has a higher effectiveness percentage than the CPRswim protocol, in which there is a considerably higher compression rate but with less efficacy.

## 1. Introduction

Cardiopulmonary resuscitation (CPR) after a life-threatening emergency is often performed in a non-resting state, with rescuers running at high intensity, swimming, carrying a victim, or acting in adverse heat or humidity conditions [1,2,3]. This uncertainty evidences the pertinence of standard life saving procedures and how to behave in different operational contexts. During cardiac arrest, brain perfusion is impaired and an irreversible neuronal process begins within 5–8 min, frequently causing cerebral oxygenation reduction and brain damage [4,5]. By diminishing the time gap between collapse and CPR, survival chances with good neurological outcome will increase [6]. Thus, in an out of hospital emergency, it is fundamental to ensure sufficient oxygenated blood flow, minimizing ischemic damage. Relevant relationships between survival outcomes and CPR quality (e.g., chest compression depth, rate and fraction) have been repeatedly displayed [7,8,9,10].

The guidelines of the American Heart Association and the European Resuscitation Council describe a 30:2 compress–ventilation ratio as standard CPR procedures for adult patients [11,12]. Chest compressions should be delivered at a 100–120 compressions/min rate and 5–6 cm depth, allowing full chest recoil and minimizing interruptions [11]. High-quality chest compression delivery is challenging and rescuers’ fatigue is a likely contributor to variability application and inflation quality during resuscitation [13]. In fact, chest compression depth can degrade after 1 min CPR (reducing continuously up to 3 min [14,15]), with rescuers frequently not being able to recognize when fatigue starts affecting their performance [16].

Rescuer interventions in a life-threatening emergency require a 3–12 min vigorous and stressful effort at intensities higher than the ventilatory threshold and up to the maximum oxygen consumption mark [17,18]. However, some efforts are shorter than 1 min, relying heavily on anaerobic energy since there was not sufficient time for recruiting aerobic energy (with maximum heart rate and maximum cardiac output being obtained latter) [17,19,20]. The influence of a typical 100 m maximal run on emergency medical technicians CPR manoeuvres has already been studied [1], but the fatigue effect of different locomotion modes on CPR quality is not sufficiently well addressed. In the current study, it was hypothesized that previous high intensity runs on the beach and swimming in the sea will induce a fatigue state that substantially decreases certified lifeguards’ CPR effectiveness. Complementarily, we also searched for the variables that discriminate the fatigue level that is acceptable for guaranteeing the correct CPR protocol application.

## 2. Materials and Methods

### 2.1. Participants

Thirty trained and certified male lifeguards (age 24.5 ± 3.9 years old, height 178.2 ± 7.4 cm, wingspan 182.1 ± 10.3 cm and body mass 76.9 ± 10.6 kg) voluntary participated in the current study. They were members of the Lifesaving Federation of Galicia, working on the beaches of La Coruña region (Spain), and offered to participate in the current study. Thus, a non-probabilistic and intentional selection of the study participants was carried out. Participants were all former swimmers and their specific knowledge plus lifeguard physical aptitudes were officially evaluated by professionals through theoretical and practical examinations (based on the recommendations described at [11,12]) < 1 year before the experiments. Rescuers were familiarized with the testing procedures prior to the experiments and were encouraged to give their best effort. However, some to the subjects were excluded of the study because they did not show well developed physical and technical skills to conduct the three experimental conditions. Before the experiments, a written informed consent to participate was obtained. The local lifesaving federation approved the study design and the experimental protocol was conducted in accordance with the Declaration of Helsinki.

### 2.2. Protocol

**Figure 1:**
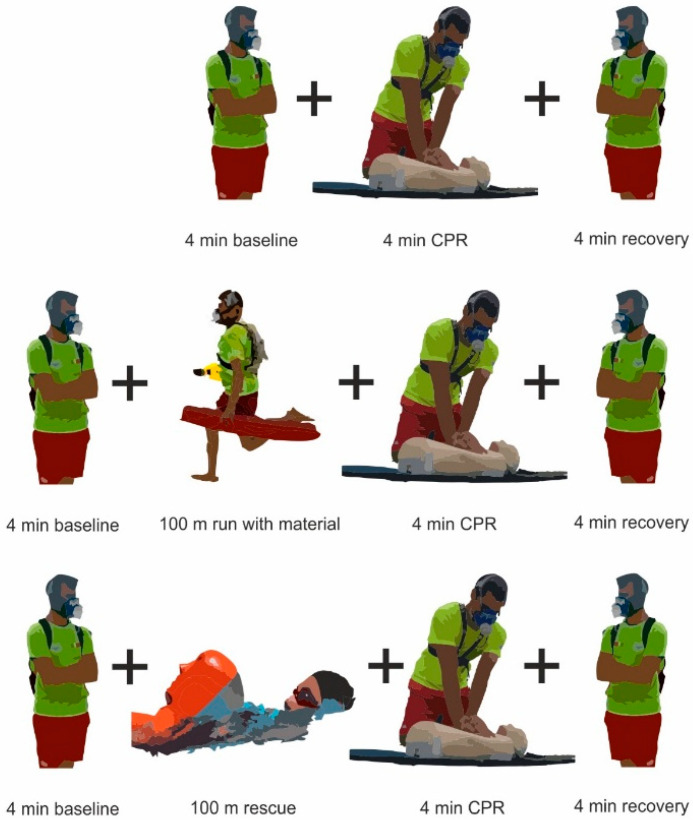
CPRbase, CPRrun and CPRswim experimental conditions (upper, middle and lower panels, respectively). The manikin used is in accordance with the American Heart Association and European Resuscitation Council guidelines for CPR practice [11,12].

Rescuers arrived at the beach 30 min before the experiments, which took place from 11:00–13:00 to avoid eventual circadian rhythm effects [21]. They were fully rested, did not engage on vigorous efforts in the prior 48 h and had an adequate nutritional intake. After dynamic stretching and low-intensity continuous running, participants were divided by chance (using envelopes) into three groups that randomly performed (with >30 min rest in-between): (i) 4 min CPR and 4 min of standing recovery (CPRbase); (ii) 100 m maximal intensity running at the beach (carrying the necessary equipment to perform basic life support aid), 4 min CPR and 4 min of standing recovery (CPRrun); and (iii) a combined water rescue simulation that involved swimming to a target, dragging an adult manikin to shore, 4 min CPR and 4 min of standing recovery (CPRswim). The water temperature was 22 °C and the wind plus wave conditions equalled 1–2 on the Beaufort scale. In the CPRbase condition, rescuers (wearing a portable gas analyzer [1,2]) performed 4 min CPR on an adult manikin (Laerdal^®^ Resusci Anne Torso; Laerdal Medical, Stavanger, Norway). Standing on their knees, they used a 30:2 compression–ventilation ratio and a 5 cm compression depth controlled by a beep signal issued by the manikin (in accordance with the European Resuscitation Council 2005 and 2015 guidelines). Although ventilations were made direct to the manikin, the use of a traditional facemask connected to the portable gas analyzer prevented the manikin from being ventilated. Then, the rescuers reassumed a standing position to recover for 4 min or until ventilatory baseline values were reached (Figure 1 illustrates the different experimental conditions).

In the CPRrun, the rescuers performed a 100 m maximal run in the sand toward a resuscitation manikin (same model described above) wearing a swimsuit, t-shirt, flip-flops and facemask connected to a portable gas analyzer, and carried flippers, a floppy and a walkie talkie. Then, 4 min CPR manoeuvres were performed and the standing position was reassumed (as previously described). In the CPRswim, rescuers were 5 m from the shore and entered the water placing their fins with their most effective technique [22]. Then, they carried out a 50 m maximal front crawl rescue to reach an adult resuscitation manikin placed on top of a jet ski board (55–60 m from the shore) and returned to the starting point swimming on their back carrying the manikin already described. After reaching the shore and being engaged with the portable gas analyzer, subjects ran 5 m as fast as they could towards another resuscitation manikin to perform 4 min CPR (as described above). After that, subjects reassumed a standing position to recover.

### 2.3. Measurement Equipment and Data Acquisition

Ventilatory variables were continuously measured breath-by-breath during baseline, CPR and recovery periods were measured using a telemetric portable gas analyzer (K4b^2^, Cosmed, Rome, Italy) placed close to the rescuers center of mass [1,2]. K4b^2^ was calibrated before each experimental condition with gases of known concentration (16% O_2_ and 5% CO_2_) using a 3 L syringe [23,24]. CPR manoeuvres were recorded using two HD video cameras (Sony, HDR PJ30VE, Sony Corporation, Tokyo, Japan), operating at a 100 Hz frequency, mounted frontal and laterally on rigid tripods (HAMA Star 63, Hama Technics S.L., Barcelona, Spain). The temporal analysis of the CPR techniques was conducted independently by two observers (with 0.96 reliability index) through a photogrammetric approach using Media Player Classic Home Cinema (MPC-HC v.1.7.10. 64 bits, Microsoft Windows, Microsoft Corporation, Redmond, WA, USA).

### 2.4. Data Filtering and Processing Prior to Analysis

Errant breaths (occurring occasionally due to coughing) and signal interruptions that do not represent the physiological functioning during exercise were excluded from the ventilatory analysis, including only those values between mean ± 4 standard deviations [2,23,24]. Then, individual breath-by-breath oxygen consumption (VO_2_) responses were smoothed using a three-breath moving average and time averaged every 10 s [2,23,24]. Physiological measures of respiratory frequency, tidal volume, minute ventilation, VO_2_, volume of carbon dioxide expired and end-tidal carbon dioxide tension were measured throughout the 4 min baseline, over each min along CPR and in the recovery period.

The time measures when checking vital signals in the first insufflation and during ventilations and compressions, as well as the compressions rate, were measured in all CPR experimental conditions. Ventilation efficacy was determined using an interval of 0.8–1.4 s since, according to the European Resuscitation Council 2015, the optimal value per ventilation is 1.1. The compression rate was measured over the first, second, third and fourth completed CPR cycles (three full cycles with insufflations and compressions each cycle), i.e., at cycles 3, 6, 9 and 12, respectively. The compression efficacy accepted the value of 30 chest compressions followed by two ventilation breaths as reference (as proposed by the European Resuscitation Council 2005). The effort intensity perception was also assessed using the Borg 0–10 scale.

### 2.5. Statistical Analyses

All variables are reported as mean ± SD and data normality and homogeneity were assessed using the Shapiro–Wilk test. A paired *t*-test analyzed the differences between CPR conditions and an ANOVA for repeated measures compared the baseline conditions and the CPR cycles (with significant effects further explored using Bonferroni post hoc procedures). All statistical procedures were conducted with SPSS 25.0 and statistical significance was set at *p* < 0.05.

## 3. Results

The time values necessary to reach the manikin in the CPRrun and CPRswim (including putting on the fins) were 25.1 ± 2.8 and 53.7 ± 8.8, respectively. In this latter condition, rescuers spent more than 99.8 ± 17.4 s returning to shore carrying the manikin and 10.5 ± 3.9 s to run 5 m toward the manikin to begin the CPR manoeuvres. Physiological data for CPRbase, CPRrun, CPRswim at baseline, CPR cycles 3, 6, 9, and 12, total CPR and recovery are shown in Table 1. Many physiological variables were higher in the CPRrun and CPRswim compared to CPRbase, with the RPE values displaying the same behaviour (4.8 ± 1.3 and 5.5 ± 1.2 vs. 2.7 ± 0.7, respectively, for a *p* < 0.001). In addition, we observed some physiological differences between baseline and cycles 3, 6, 9, 12 and Total CPR for each experimental condition, i.e., CPRbase, CPRrun and CPRswim.

The technical related variables values for the different experimental conditions at cycles 3, 6, 9 and 12 and total CPR are displayed in Table 2. No differences were found in the time spent to complete three full cycles in any of the cycles (except for cycle 6 that presented lower values in CPRSwim than in the other conditions). Compressions rate in the CPRSwim was higher than in CPRrun and CPRbase in all cycles, and total CPR, and presented similar values in CPRrun and CPRbase conditions (except for cycle 9). Ventilation efficacy was always higher in the conditions preceded by intense physical exertions than at CPRbase, with differences between the CPRrun and CPRswim only for cycle 3 s. Compression efficacy values in CPRbase were always higher than CPRrun and higher than CPRswim for cycles 3 and 9 and total CPR (CPRrun and CPRswim differed in cycles 3, 6 and 12).

## 4. Discussion

The current research aimed to observe if typical locomotion modes used by lifeguards for approaching the victim would induce a fatigue state that might compromise CPR effectiveness. This followed previous studies conducted in different contexts and with other professionals that used these lifesaving techniques (e.g., [1,2,3]). Our hypothesis was partially verified since the performed run and swim intense exertions had a strong physiological impact on CPR, evidencing higher cardiopulmonary values in CPRrun and CPRswim than CPRbase. In addition, pre-CPR vigorous exercise had an evident negative influence on CPR effectiveness (even if no relevant changes were observed for ventilation efficacy and compression rate). The current data suggest that after maximal run or swimming rescue, the overall CPR protocol seems not to be jeopardized, but the ability to maintain good CPR technical quality is affected. Complementarily, when looking for variable(s) that can discriminate what level of previous fatigue is acceptable to guarantee the correct CPR protocol application, some specific cardiopulmonary, RPE and technical variables were identified.

The CPRrun and CPRswim intense physical efforts had an evident repercussion in rescuers’ physiologic state (compared to CPRbase). Respiratory quotient, by estimating substrate oxidation through the relationship between VCO_2_ produced and VO_2_ consumed, is one physiological variable that can characterize exercise intensity well [25]. Its mean value remained at <1.0 throughout the total CPR (except for the cycle 3) in CPRbase, whereas it was situated between 1.03 and 1.25 in the CPRrun and CPRswim. Moreover, tidal volume was higher in CPRrun and CPRswim compared with CPRbase and, despite the short time necessary to run the 100 m to reach the manikin and the longer time necessary to carry out the in-water rescue, it presented a similar response in these conditions. The cardiopulmonary stress induced by previous intense exercise was also evidenced by the higher respiratory frequency at recovery, as well as by the elevated RPE in the CPRrun and CPRswim compared to CPRbase.

Of the evaluated cardiopulmonary outcomes, tidal carbon dioxide showed similar total CPR values in CPRbase and CPRswim, and higher values for CPRrun. This variable ranges from −24 to 27 mmHg in patients with in- and out-of-hospital cardiac arrest and is independent of compression rate [26]. When ventilation is held constant, ideally both in ventilation rate and tidal volume, tidal carbon dioxide becomes an excellent measure of pulmonary blood flow [27]. In the current study, even if not directly measured in the manikin but through the rescuers expired air, the observed tidal carbon dioxide values suggest that an adequate quality CPR was provided since a cut point of 25.5 mmHg was established for initial tidal carbon dioxide [28]. In fact, for patients with initial values of <25.5 mmHg, survival benefits ceased at an earlier point in resuscitation, whereas above this threshold the probability of survival cumulatively increased for a longer period. Optimal tidal carbon dioxide depends also on chest compression quality, ventilation rate and tidal volume [29] (all deteriorating with fatigue) and were higher in cycles 3, 6, 9, and 12 of the CPRrun (although no difference was observed in the total course).

In the current study, the CPRbase mean VO_2_ and HR values represented a low physiological demand, with similar rates being previously reported [27,28]. Even if emergency medical settings mostly involve being at rest, occasionally, these technicians need to handle CPR in a limited time after running at a high intensity to reach to the victim as fast as possible. The cardiovascular demands associated with such specific tasks ranged from 13.3 to 27.2 mL·kg^−1^·min^−1^ and 144 to 155 bpm, and are close to different exercise modes performed at continuous moderate intensity (60–70% of maximum intensity) [20]. In healthy subjects, this is the highest work percentage recommended to avoid excessive use of the anaerobic metabolism and consequent installation of fatigue. Considering that the rescuers’ energetic demands depend upon the specific role assumed (e.g., performing on a flat surface, steep slope, or stairs), their occupational health training should include a variety of exercises covering potential incident situations.

International resuscitation guidelines recommend that chest compressions should be delivered at a rate of at least 100–120 compressions/min [11], with prompt CPR delivery assuming an essential role in the survival chain for cardiac arrest resuscitation. In fact, it was recently shown that the return of spontaneous circulation from in-hospital cardiac arrest was associated with higher chest compression rates, which is consistent with out-of-hospital CPR quality related studies [26,30]. However, the −87 and 95 compressions/min observed by us for CPRbase and CPRrun were slightly lower than the gold standard, which is in line with studies that reported 85–100 chest compressions/min in survivors (compared with non-survivors who received a lower rate) [31]. In the current study, the compression rate over the first three complete cycles was lower than the subsequent three CPR complete cycles for the three experimental conditions, undervaluing its values over the total course. In fact, the compression rate at second, third and fourth three complete CPR (cycles 6, 9 and 12, respectively) in all conditions were very close to those recommend by the international resuscitation guidelines [11].

The above suggests that the CPR manoeuvres conducted immediately after intense running and swimming were not moderate exertions but efforts situated in the heavy intensity domain. When comparing exercise modes, a better Total CPR compressions rate in CPRswim was observed, in line with previous data that evidenced post-rescue correct CPR manoeuvres even when the compression rate diminished [31]. In fact, we have also observed an inverse behaviour between compression rate and compression efficacy (in the three experimental conditions), expressing that these higher compressions rate values do not translate into greater compression efficacy. Moreover, all the CPRrun and CPRswim compressions efficacy values were lower than those obtained at CPRbase, suggesting that, in contrast to what was observed for the physiological variables, mechanical-related fatigue might play an important role in CPR efficacy.

The ideal time per ventilation is also a fundamental aspect to consider as a reference when conducting CPR, since it is a guideline from the European Resuscitation Council 2015 that assures that the correct air flow is guaranteed to the injured person. Our current data indicate low percentages of ventilation efficacy for the three experimental conditions, but performing CPRrun and CPRswim significantly increased CPR ventilation technical-related variables in our trained certified lifeguards (compared to the CPRbase). Different studies suggest the use of ventilation devices (e.g., bag-valve-mask [32,33]) to avoid contagion and guarantee the correct air flow during basic life support [34,35,36].

Although the current study’s experimental conditions were very close to what happens in real life after a life-threatening emergency, the fact that the lifeguards were not facing a true accident can be considered a study constraint. Another limitation was the impossibility of using the K4b^2^ gas analyzer during the water rescue, not allowing us to observe the ventilatory data behaviour from the swimming effort. This fact, as well as the use of more sophisticated CPR manikins that can give other relevant and reliable variables, should be tried to be implemented in future studies on the topic. Lastly, since fatigue negatively influenced the CPR quality, the training process for certified rescuers (and their posterior continuous training along the years) should take this into consideration, helping rescuers to cope with emergency situations. Finally, the use of ventilation devices during CPR should be included in the training process, aiming to improve professionals’ ventilatory efficacy and corresponding survival outcomes.

## 5. Conclusions

The CPRrun and CPRswim protocols induced relevant physiological stress in each CPR cycle and in the overall CPR manoeuvres. Both CPRrun and CPRswim were conducted in the heavy exercise intensity domain despite the higher physiological values obtained after running (even if a higher rate of perceived effort was pointed out in CPRswim). Previously induced fatigue was reflected in the CPR quality, with lower ventilatory efficacy values being found in all experimental conditions. Even though better compression rate records have been found in CPRrun and CPRswim, the compression efficacy values suggest that fatigue negatively influenced the CPR quality. The probability of saving lives with a better management of efforts is a significant contribution of the current study, requiring a greater and better knowledge of the management of the physical capacities of lifeguards in rescue situations.

## Figures and Tables

**Table 1:** Mean ± SD values for the cardiopulmonary variables assessed during baseline, CPR cycles, total CPR and recovery in the different experimental conditions.

Variables		Rf (b·min^−1^)	TV (l)	V_E_ (L·min^−1^)	VO_2_ (mL/kg/min)	R	VCO_2_ (mL·min^−1^)	HR (bpm)	PetCO_2_ (mmHg)
CPRbase	Baseline	16.6 ± 4.3	1.2 ± 0.5	18.0 ± 3.6	11.9 ± 3.8	0.95 ± 0.2	0.49 ± 0.1	88 ± 13	33.6 ± 4.0
	Cycle 3	28.7 ± 8.1 ^1^	1.4 ± 0.4	36.3 ± 7.8 ^1^	14.1 ± 5.3 ^1^	1.14 ± 0.2 ^1^	0.96 ± 0.3 ^1^	112 ± 15 ^1^	29.5 ± 4.2 ^1^
	Cycle 6	29.1 ± 8.5 ^2^	1.5 ± 0.4 ^2^	39.7 ± 9.1 ^2^	14.9 ± 6.3 ^2^	0.86 ± 0.2 ^2^	1.13 ± 0.3 ^2^	114 ± 14 ^2^	31.6 ± 6.2 ^2^
	Cycle 9	27.7 ± 7.0 ^3^	1.6 ± 0.4 ^3^	43.2 ± 9.3 ^3^	13.1 ± 4.4 ^3^	0.88 ± 0.2 ^3^	1.26 ± 0.3 ^3^	114 ± 15 ^3^	32.9 ± 5.6
	Cycle 12	27.6 ± 7.1 ^4^	1.6 ± 0.5 ^4^	42.4 ± 9.9 ^4^	11.6 ± 8.0	0.90 ± 0.2 ^4^	1.21 ± 0.3 ^4^	114 ± 16 ^4^	33.1 ± 4.4
	Total CPR	28.2 ± 7.6 ^5^	1.5 ± 0.4 ^5^	40.4 ± 9.4 ^5^	13.5 ± 6.2 ^5^	0.94 ± 0.2 ^5^	1.14 ± 0.3 ^5^	114 ± 15 ^5^	31.7 ± 5.3 ^5^
	Recovery	19.2 ± 4.9	1.4 ± 0.4	24.8 ± 4.6	14.1 ± 5.2	0.91 ± 0.2	0.70 ± 0.2	95 ± 16	34.1 ± 3.6
CPRrun	Baseline	16.8 ± 4.4	1.3 ± 0.6	17.9 ± 3.5	12.2 ± 3.6	0.96 ± 0.2	0.49 ± 0.2	89 ± 15	33.9 ± 3.8
	Cycle 3	30.1 ± 3.8	2.7 ± 0.5 *	81.7 ± 16.3 ^1^	22.3 ± 9.8 ^1^	1.14 ± 0.3	2.83 ± 0.9 ^1^	147 ± 16 ^1^	39.2 ± 7.7 ^1^
	Cycle 6	29.5 ± 4.0	2.6 ± 0.5 *	76.6 ± 17.9 ^2^	25.5 ± 8.3 ^2^	1.25 ± 0.3 ^2^	2.35 ± 0.8 ^2^	137 ± 18 ^2^	36.5 ± 7.7 ^2^
	Cycle 9	30.4 ± 4.7 ^3^	2.3 ± 0.5 *	70.3 ± 17.2 ^3^	23.5 ± 7.7 ^3^	1.18 ± 0.3 ^3^	2.04 ± 0.6 ^3^	132 ± 19 ^3^	35.3 ± 3.5 ^3^
	Cycle 12	29.9 ± 4.9	2.3 ± 0.5 *	66.3 ± 15.9 ^4^	20.7 ± 13.1 ^4^	1.15 ± 0.3 ^4^	1.85 ± 0.5 ^4^	130 ± 17 ^4^	34.7 ± 3.7
	Total CPR	29.9 ± 4.3 ^5^	2.5 ± 0.5 *	73.8 ± 17.7 *^,5^	23.0 ± 9.9 *^,5^	1.18 ± 0.3 *^,5^	2.27 ± 0.8 *^,5^	137 ± 19 *^,5^	36.4 ± 6.2 *^,5^
	Recovery	22.5 ± 5.2 *	1.7 ± 0.5 *	37.5 ± 9.5 *	22.7 ± 8.7 *	1.05 ± 0.2 *	1.01 ± 0.3 *	111 ± 15 *	33.5 ± 3.3
CPRswim	Baseline	16.7 ± 4.2	1.3 ± 0.4	17.7 ± 3.4	11.7 ± 3.6	0.96 ± 0.4	0.50 ± 0.1	92 ± 16	34.0 ± 3.9
	Swim	21.8 ± 4.4 *^,†^	1.0 ± 0.3 *^,†^	93.4 ± 23.5 *^,†^	19.1 ± 9.1 *^,†^	1.19 ± 0.1 *^,†^	2.94 ± 0.7 *^,†^	77 ± 15 *	30.01 ± 1.7 *
	Cycle 3	33.8 ± 6.3 ^1,†^	2.8 ± 0.7 ^1^	93.4 ± 23.5 ^1,†^	19.8 ± 7.7 ^1^	1.19 ± 0.1	2.58 ± 0.6 ^1^	152 ± 15 ^1^	37.02 ± 5.0 ^1,†^
	Cycle 6	33.9 ± 5.1 ^2,†^	2.7 ± 0.6 ^2^	91.3 ± 18.2 ^2^	20.5 ± 8.3 ^2^	1.25 ± 0.2 ^2^	2.03 ± 0.3 ^2^	144 ± 18 ^2^	32.3 ± 4.9 ^†^
	Cycle 9	33.3 ± 5.9 ^3,†^	2.4 ± 0.5 ^3^	79.4 ± 14.8 ^3^	19.8 ± 9.2 ^3^	1.19 ± 0.1 ^3^	1,77 ± 0.3 ^3^	138 ± 17 ^3^	30.9 ± 4.1 ^3,†^
	Cycle 12	33.2 ± 6.3 ^4^	2.3 ± 0.5 ^4^	73.6 ± 13.6 ^4^	23.3 ± 10.6 ^4^	1.03 ± 0.1	1.58 ± 0.4 ^4^	134 ± 16 ^4^	28.8 ± 3.9 ^4,†^
	Total CPR	31.8 ± 4.4 *^,5^	2.2 ± 0.5 *^,5^	67.9 ± 14.8 *^,5^	20.6 ± 9.1 *^,5^	1.16 ± 0.1 *^,5^	2.18 ± 0.7 *^,5^	133 ± 15 *^,5^	30.0 ± 4.7 *^,5^
	Recovery	22.5 ± 3.9 *	1.7 ± 0.4 *	37.4 ± 10.1 *	18.8 ± 8.9 *	1.01 ± 0.1 *	0.80 ± 0.2 *^,†^	108 ± 14 *	26.3 ± 2.9 *^,†^

Respiratory frequency (Rf), tidal volume (TV), minute ventilation (VE), volume of oxygen consumed (VO_2_), respiratory quotient (R), volume of carbon dioxide expired (VCO_2_), heart rate (HR) and tidal carbon dioxide (PetCO_2_). * and ^†^: different from CPRbase and CPRrun (respectively) for <0.05; ^1^, ^2^, ^3^, ^4^ and ^5^: differences between baseline and cycles 3, 6, 9, 12 and Total CPR for each experimental condition.

**Table 2:** Mean ± SD values for the technical variables assessed during CPR cycles (3, 6, 9 and 12) and total CPR in the CPRbase, CPRrun and CPRswim conditions.

Variables		Duration (s)	Compressions Rate (n/min)	Ventilation_ef_ (%)	Compression_ef_ (%)
CPRbase	Cycle 3	72.2 ± 13.5	77.6 ± 11.9	32.6 ± 40.1	81.1 ± 34.7
	Cycle 6	60.8 ±13.4	92.6 ± 15.6	39.1 ± 39.9	78.9 ± 35.5
	Cycle 9	56.9 ± 6.8	96.1 ± 11.6	38.4 ± 39.1	75.6 ± 33.8
	Cycle 12	44.6 ± 13.1	97.4 ± 10.8	23.9 ± 34.2	41.1 ± 28.0
	Total CPR	240.2 ± 2.8	87.4 ± 14.8	33.5 ± 38.3	69.2 ± 28.0
CPRrun	Cycle 3	72.5 ± 8.9	76.5 ± 10.1	47.8 ± 42.0 *	68.9 ± 36.0 *
	Cycle 6	58.5 ± 7.9	92.5 ± 12.7	52.2 ± 42.6 *	68.9 ± 36.0 *
	Cycle 9	58.8 ± 7.9	93.4 ± 11.8 *	55.8 ± 42.2 *	67.8 ± 36.6 *
	Cycle 12	43.8 ± 12.4	96.2 ± 9.0	42.0 ± 42.4 *	47.8 ± 29.5 *
	Total CPR	235.3 ± 28.2	86.9 ± 10.4	49.5 ± 42.3 *	63.3 ± 29.5 *
CPRswim	Cycle 3	69.9 ± 13.1	83.0 ± 13.2 *^,^^†^	55.9 ± 40.7 *^,^^†^	63.3 ± 37.5 *^,^^†^
	Cycle 6	55.3 ± 9.0 *^,^^†^	103.2 ± 15.6 *^,^^†^	54.8 ± 41.8 *	76.7 ± 32.9 ^†^
	Cycle 9	56.5 ± 9.8	100.9 ± 15.8 *^,^^†^	53.8 ± 41.9 *	67.8 ± 36.6 *
	Cycle 12	42.2 ± 12.9	101.6 ± 15.6 *^,^^†^	43.0 ± 39.6 *	41.1 ± 39.8 ^†^
	Total CPR	240.7 ± 2.7	95.5 ± 15.8 *^,^^†^	51.9 ± 41.0 *	62.2 ± 28.3 *

Ventilation efficacy (Ventilationef) and compression efficacy (Compressionef). * and ^†^: different from CPRbase and CPRrun, respectively, for <0.05.

## Data Availability

Raw data can be requested by sending an email to abraldes@um.es.
